# Rhino-Orbital Mucormycosis Associated With COVID-19

**DOI:** 10.7759/cureus.10726

**Published:** 2020-09-30

**Authors:** Salil Mehta, Abha Pandey

**Affiliations:** 1 Ophthalmology, Lilavati Hospital and Research Center, Mumbai, IND; 2 Chest Medicine, Lilavati Hospital and Research Center, Mumbai, IND

**Keywords:** mucormycosis, orbit, covid-19, fungal infections

## Abstract

Coronavirus disease 2019 (COVID-19) infections may be associated with a wide range of bacterial and fungal co-infections. We report the case of a patient with COVID-19 infection, which, during the course of the treatment, developed rhino-orbital mucormycosis. A 60- year-old male patient, a longstanding diabetic, with a positive reverse-transcriptase polymerase chain reaction (RT-PCR) for severe acute respiratory syndrome coronavirus 2 (SARS-CoV-2), was admitted for treatment. He received parenteral meropenem and oral oseltamivir with parenteral methylprednisolone. Over the course of the admission, he developed signs of orbital cellulitis. Magnetic resonance imaging (MRI) of the brain, orbits, and paranasal sinuses, revealed soft tissue swelling in the right preseptal, malar, premaxillary and retrobulbar regions with paranasal sinusitis. A nasal biopsy revealed broad aseptate filamentous fungal hyphae suggestive of mucormycosis, which was confirmed on culture. Extensive use of steroids/monoclonal antibodies/broad-spectrum antibiotics may lead to the development/exacerbation of a preexisting fungal disease. Physicians should be aware of the possibility of secondary invasive fungal infections in patients with COVID-19 infection.

## Introduction

The coronavirus disease 2019 (COVID-19) infection caused by the novel severe acute respiratory syndrome coronavirus 2 (SARS-CoV-2) may be associated with a wide range of disease patterns, ranging from mild to life-threatening pneumonia. A wide range of bacterial and fungal co-infections may exist and may be associated with preexisting morbidity (diabetes mellitus, lung disease) or may develop as a hospital-acquired infection such as ventilator-associated pneumonia. India has a high prevalence rate of type 2 diabetes mellitus (8.9% of adults, 77 million patients), which is a well-known risk factor [1]. We report the case of a patient with COVID-19 infection, who during the course of the treatment, developed rhino-orbital mucormycosis.

## Case presentation

A 60-year-old male patient was admitted with a three-day history of severe breathlessness, pyrexia, tachypnea, and generalized malaise. He was a longstanding diabetic (> 10 years) on oral antihypoglycemic tablets. On examination, his pulse rate was 80/minute, blood pressure was 150/90 mmHg, he was afebrile on admission, respiratory rate was 26/minute, with a specific oxygen saturation of 86% on oxygen supplementation (10 liters/min). The relevant physical examination revealed bilateral crepts at the lung bases with a normal cardiovascular and neurological exam. A non-healing ulcer consistent with the diabetic peripheral vascular disease was seen on his right foot.

**Figure 1:**
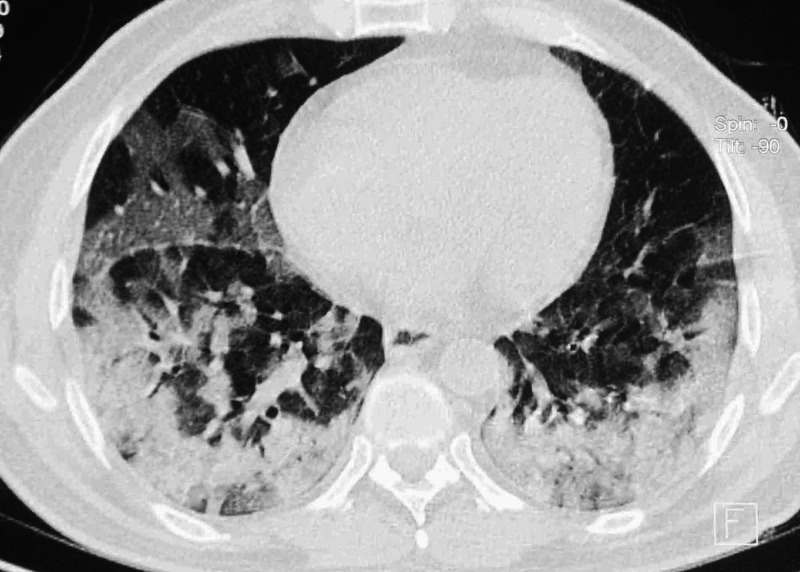
Computed tomography (CT) scan of the chest CT scan showing extensive peripheral ground-glass opacities in both lungs

A reverse-transcriptase polymerase chain reaction (RT-PCR) from a nasopharyngeal swab was positive for the SARS-CoV-2 virus. A computed tomography (CT) scan of the chest showed multiple patchy ground-glass opacities in both lungs involving both upper lobes, the right middle lobe, and the lingula predominantly in a peripheral distribution strongly suggestive of COVID-19 infection (Figure 1).

He was started on intravenous meropenem (1 gm thrice daily), oral oseltamivir (75 mg twice daily), with intravenous methylprednisolone (40 mg twice daily) and dexamethasone (4 mg twice daily), in conformity with the local protocol, along with general supportive care. His diabetes mellitus was managed with insulin adjusted as per a sliding scale based on his premeal blood sugar levels adjusted to maintain 180-200 mg/dl. He also received subcutaneous enoxaparin (40mg/0.4 ml) twice daily.

He gradually deteriorated with the onset of acute respiratory distress syndrome over the next few days. On day three, he was shifted to non-invasive ventilation to maintain his oxygen saturation. On day four, he received a single dose of injectable tocilizumab (400 mg) and was started on an oral combination of sitagliptin/metformin (50/500) twice daily and oral metformin (500 mg) thrice daily with subcutaneous insulin glargine (20 units) at night with regular insulin as needed to continue to maintain a blood sugar level of 180-200 mg/dl.

**Figure 2:**
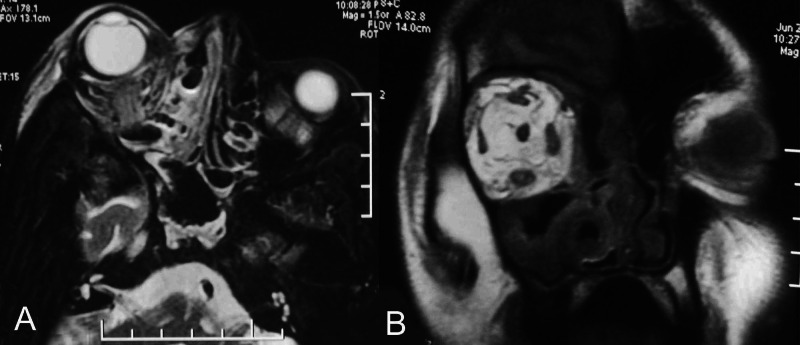
Magnetic resonance imaging of the orbit (A) Axial MRI (T2) showing hyperintensity of the ethmoid and nasal sinus and of the retrobulbar space (B) coronal section (T1) showing irregular hyperintensity of the retrobulbar space

On day 10, bilateral lid edema with right eye prominence was noted and topical moxifloxacin was prescribed. The next day, on MRI of the brain, orbits, and paranasal sinuses, a soft tissue swelling was noted in the right preseptal, malar, premaxillary, and retrobulbar regions, which appeared hyperintense on T2 and fluid-attenuated inversion recovery (FLAIR). The right extraocular muscles were bulky and mild right proptosis was noted. Sinusitis in the form of significant mucosal thickening in the right frontal, maxillary, and ethmoidal sinus was also seen (Figure 2).

A preliminary diagnosis of right orbital cellulitis was made and he was transferred to a tertiary care center. On admission, he was afebrile, breathless, and hypoxic. He underwent a complete systemic and laboratory evaluation. Relevant baseline investigations revealed a hemoglobin value of 10.40 gm/dl (normal 13-17 gm/dl), mild lymphopenia (9.60%; normal 20-40%), elevated serum creatinine (1.57 mg/dl; normal 0.70-1.20). C-reactive protein (CRP) was 29.53 mg/l (normal <5.0), procalcitonin (PCT) was 0.34 ng/ml (normal <0.5), with a D-dimer assay of 1547 ng/ml (normal 0-243) and an IL6 level of 3439 micrograms/ml (normal 0-7.0), suggestive of a severe cytokine storm.

He was continued on intravenous meropenem (1 gm thrice daily) and vancomycin (1 gram twice daily) with the addition of amphotericin B (0.5 mg/kg/day) and the cessation of steroid therapy.

An ophthalmic evaluation was requested the next day. On examination, the right eye was mildly proptotic with extensive areas of edema in the periorbital region with soft tissue necrosis along the medial half of the upper and lower lids. The right eye was congested with conjunctival edema and signs of exposure keratitis. The left eye appeared fixed and had a dilated non-reactive pupil (to light) either due to extension of infection to the other cavernous sinus or due to COVID-19 coagulopathy. Visual acuity and detailed ocular movements could not be assessed, as the patient was drowsy and not responsive. The clinical picture and MRI picture strongly suggested invasive fungal infection likely mucormycosis. A nasal swab was non-contributory but a nasal biopsy from the middle turbinate revealed broad aseptate filamentous fungal hyphae suggestive of mucormycosis, which was confirmed on a Sabourauds Dextrose Agar culture.

The patient continued to deteriorate, was ventilated, and eventually required inotropic support. Due to persistent hypotension, we were unable to carry out repeat imaging or any debridement measures. Despite all measures, he died on day six of this admission.

## Discussion

A complex interplay of factors, including preexisting diseases, such as diabetes mellitus, previous respiratory pathology, use of immunosuppressive therapy, the risk of hospital-acquired infections, and systemic immune alterations of COVID-19 infection itself may lead to secondary infections, which are increasingly being recognized in view of their impact on morbidity and mortality [2]. In a recent review, 62/806 (8%) patients had secondary bacterial or fungal infections during hospital admission. There was widespread use of broad-spectrum antibiotics, with as many 1450/2010 (72%) of patients receiving these drugs, often with no underlying evidence of infection [3].

Current guidelines in India recommend intravenous methylprednisolone 0.5-1 mg/kg/day for three days in moderate cases and 1-2 mg/kg/day in severe cases [4]. The National Institute of Health recommends the use of dexamethasone (6 mg per day for a maximum of 10 days) in patients who are ventilated or require supplemental oxygen but not in milder cases [5]. The guidelines specifically mention the risk of developing a secondary infection [6].

There are specific pathophysiologic features of COVID-19 that may permit secondary fungal infections, including a propensity to cause extensive pulmonary disease and the subsequent alveolo-interstitial pathology that may enhance the risk of invasive fungal infections. Second, the immune dysregulation associated with COVID-19, with reduced numbers of T lymphocytes, CD4+T, and CD8+T cells, may alter innate immunity [7].

In one cluster from New Delhi, India, 15 admitted patients with COVID-19 infection developed bloodstream candida infections. Of these, 10 had a Candida auris infection, of whom six died (60%) [8]. White et al. screened 135 adults with COVID-19 infection and reported an incidence of invasive fungal infections of 26.7% (commonly aspergillosis (14.1%), or yeast, usually candida (12·6%)). Patients with invasive fungal diseases had higher mortality (53% with vs 31% without), which was significantly reduced by appropriate therapy. Corticosteroid therapy and a past history of chronic pulmonary disease were associated with a higher risk of invasive fungal disease [9]. Similarly, high incidences have been seen in Pakistan (23/147, 15.6%) and Italy (30/108, 27.7%), with the authors suggesting that the development of invasive fungal infections alters the natural history of the disease [10-11]. Song et al. have suggested an algorithm for the early diagnosis and management of common invasive fungal infections (aspergillus, candidiasis, cryptococcosis, and mucormycosis) [12].

We conducted a literature search on Pubmed (www.pubmed.gov) with the search terms “mucormycosis,” “mucor”, AND “COVID-19” to identify any additional reported cases. Only a limited number of cases of secondary mucormycosis have been previously reported. Hanley et al. have reported a case of a 22-year-old male with COVID-19 pneumonia and a middle cerebral artery infarct in whom disseminated mucormycosis involving the lungs and brain was incidentally discovered during a postmortem study [13].

Werthman-Ehrenreich reported the case of a 33-year-old female who presented with left-sided ptosis and proptosis with altered sensorium. Investigations revealed diabetic ketoacidosis with COVID-19 infection. Facial imaging was significant for maxillary and ethmoidal sinus mucosal thickening. An MRI of the brain showed multiple areas of infarction and ischemia indicating invasive fungal disease. Mucor was demonstrated via a nasal biopsy and subsequent culture. The author suggests that early identification of fungal co-infections may significantly reduce morbidity and mortality [14].

The patient we describe with severe COVID-19 was a long-standing diabetic as evidenced by his diabetic foot ulcers. The signs of orbital infection were noticed only after 10 days of admission for COVID-19 infection during which time he was treated with both broad-spectrum antibiotics and steroids. All these factors tend to facilitate fungal coinfection, along with any possible COVID-19 pathophysiological mechanisms. In our case, either a previously undiagnosed mucor infection may have been aggravated or it may have subsequently developed.

## Conclusions

COVID-19 is associated with a significant incidence of secondary infections, both bacterial and fungal probably due to immune dysregulation. Additionally, the widespread use of steroids/monoclonal antibodies/broad-spectrum antibiotics as part of the armamentarium against COVID-19 may lead to the development/exacerbation of preexisting fungal diseases. Physicians should be aware of the possibility of invasive secondary fungal infections in patients with COVID-19 infection especially in patients with preexisting risk factors and should enable early diagnosis and treatment with the subsequent reduction of mortality and morbidity. The use of therapeutic agents should be monitored to achieve a therapeutic effect at the lowest dose and shortest durations. The use of broad-spectrum antibiotics, especially in the absence of infection, should be re-evaluated.
